# Epidemiology and outcomes of out-of-hospital cardiac arrest in a developing country-a multicenter cohort study

**DOI:** 10.1186/s12873-016-0093-2

**Published:** 2016-07-28

**Authors:** Minaz Mawani, Muhammad Masood Kadir, Iqbal Azam, Amber Mehmood, Bryan McNally, Kent Stevens, Rozina Nuruddin, Mohammad Ishaq, Junaid Abdul Razzak

**Affiliations:** 1 Department of Medicine, Aga Khan University, First floor, Faculty Offices Building, Stadium road, P.O. Box 3500, Karachi, 74800 Pakistan; 2 Department of Community Health Sciences, Aga Khan University, Karachi, Pakistan; 3 International Health Johns Hopkins Bloomberg School of Public Health, Baltimore, MD USA; 4 Emory University School of Medicine & Rollins School of Public Health, Atlanta, GA USA; 5 Department of Surgery Johns Hopkins University School of Medicine, Baltimore, MD USA; 6 Karachi Institute of Heart Diseases, Karachi, Pakistan; 7 Department of Emergency Medicine, Aga Khan University & Aman Health, Aman Foundation, Karachi, Pakistan

**Keywords:** Out-of-hospital cardiac arrest, Survival, Chain-of-survival

## Abstract

**Background:**

Out-of-hospital cardiac arrest (OHCA) is one of the leading causes of death and disability worldwide. Overall survival after an OHCA has been reported to be poor and limited studies have been conducted in developing countries. We aimed to investigate the rates of survival from OHCA and explore components of the chain of survival in a developing country.

**Methods:**

We conducted a multicenter prospective cohort study in the emergency departments (ED) of five major public and private sector hospitals of Karachi, Pakistan from January 2013 to April 2013. Twenty-four hour data collection was performed by trained data collectors, using a structured questionnaire. All patients ≥18 years of age, presenting with OHCA of cardiac origin, were included. Patients with do-not-resuscitate status or referred from other hospitals were excluded. Our primary outcome was survival of OHCA patients at the end of ED stay.

**Results:**

During the three month period, data was obtained from 310 OHCA patients. The overall survival to ED discharge was 1.6 % which decreased to 0 % at 2-months after discharge. More than half (58.3 %) of these OHCA patients were brought to the hospital in a non-EMS (emergency medical service) vehicle i.e. public or private transportation. Patients utilizing non-EMS transportation reached the hospital earlier with a median time of 23 min compared to patients utilizing any type of ambulances which had a delay of 7 min hospital reaching time (median time 30 min). However, patients utilizing ambulances with life-support facilities, as compared to all other types of pre-hospital transportation, had the shortest time to first life-support intervention (15 min).

Most of the patients (92.9 %) had a witnessed cardiac arrest out of which only a small percentage (2.3 %) received bystander CPR (cardio pulmonary resuscitation). Median time from arrest to receiving first CPR was 20 min. Only 1 % of patients were found to have a shockable rhythm on first assessment.

**Conclusion:**

This study showed that the overall survival of OHCA is null in this population. Lack of bystander CPR and weaker emergency medical services (EMS) leading to a delay in receiving life-support interventions were some of the important observations. Poor survival emphasizes the need to standardize EMS systems, initiate public awareness programs and strengthen links in the chain of survival.

**Electronic supplementary material:**

The online version of this article (doi:10.1186/s12873-016-0093-2) contains supplementary material, which is available to authorized users.

## Background

Out-of-hospital cardiac arrest (OHCA) is one of the leading causes of death and disability worldwide and contributes to as high as 10 % of the total mortality in developing countries [1–3]. Adoption of large-scale public health measures targeting early interventions has resulted in a slow and gradual improvement in survival rates, but with high levels of disparities [1, 2, 4], even in developed countries like United States the average rate of survival to hospital discharge varies from <1 % to over 25 % [4, 5]. Even within the same city, there are differences of up to 40 % in and between different races [4, 5]. The OHCA survival rates show considerable variations among different continents as well (Europe 9 %, North America 6 % and Australia 11 %) [4, 6]. The variability in the survival by different regions, races and continents underscores the potential opportunities for significant improvements in the key predictors of OHCA such as provision of immediate bystander CPR (cardiopulmonary resuscitation), early defibrillation, early emergency medical services (EMS) response, and post resuscitation care [4, 6–10]. The survival differences, to a large extent, are determined by the strength of the continuum of integrated life-saving steps, often called the “chain of survival” [11].

Over 80 % of the burden of cardiac diseases is in low and middle income countries, yet incidence rates and survival from OHCA in these countries remain largely undefined [12]. Poor emergency systems, lack of focus on non-communicable diseases and inadequate medical records are some of the main reasons [13–15]. Data, where it exists, is either derived from a single hospital, characterized by varying case definitions of cardiac arrest and survival or consists of incomplete pre-hospital care details and follow-up information. In addition, very few studies have been conducted to investigate the frequency of individual components of the chain of survival and their association with immediate and long term outcomes [16–19].

The primary objective of this study was to estimate the rate of OHCA survival to ED (emergency department) discharge and to measure the critical components of the chain of survival following an OHCA in a developing country.

## Methods

### Study design and setting

This prospective cohort study was carried out in the emergency departments (ED) of five major referral hospitals of Karachi, Pakistan. Karachi is the largest city of Pakistan and the third largest city in the world by population within city limits with a most recent estimated population of 23.5 million [20, 21]. Four hospitals belonged to the public sector while one was private not-for-profit hospital. These hospitals receive patients from all parts of the city. The reasons for selecting these hospitals were; first these are the largest teaching hospitals of the city which receive majority of the EMS visits. Secondly, together these five hospitals cater a diverse population consisting of a wide range of socioeconomic status and ethnicities. In this way we were able to obtain a sample that is largely representative of the general population in Karachi and avoid any selection biases. These hospitals not only cater the population of Karachi but also patients coming from areas outside the city. The total number of hospitals in Karachi is difficult to estimate, however, these 5 represent over 35 government and private sector teaching hospitals [22–24]. Table 1 shows details of these hospitals [25–30]. Information on daily emergency department visits was obtained from hospital records.

**Table 1:** Description of hospitals, where the study was conducted

Hospitals	Sector	Number of beds in hospital	ED visits/day	Facilities of advanced cardiac interventions
Hospital A	Public	850	600	No
Hospital B	Public	1900	550	No
Hospital C	Public	1185	1000	No
Hospital D	Private	599	140	Yes
Hospital E	Public	370	154	Yes

Unlike developed EMS systems, where a single network is established to provide emergency services with a universal emergency number (e.g. 911), pre-hospital care and transportation in Pakistan is still in the developing phase. Karachi has several ambulance services, out of which only one has trained and credentialed medical staff, life-saving equipment, medical oversight and regularly monitored quality indicators. There are several other philanthropic organizations providing “emergency transportation” but without any medical intervention at the scene or during transportation [31]. For this study, type of pre-hospital transportation was categorized into three groups; ‘Ambulance with life-support interventions’ refers to ambulance services with facilities of CPR, life-saving drugs, defibrillator and a medical professional trained to deal with emergencies, ‘Ambulances without life-support interventions’ refers to those ambulances that provide early transfer to a medical facility without provision of any life-support interventions on the way, whereas, non-EMS transportation refers to any private or public transportation, other than ambulance, that is used to transfer patients to the hospital.

The Aga Khan University ethics review committee approved this study. Permission and reviews were also obtained from the head of institution/departments of all participating hospitals and their respective institutional review boards wherever available. Informed consent was obtained from the family member accompanying patients to the hospital.

### Selection of participants

This study included all patients ≥18 years presenting with OHCA to the selected emergency departments from January 22, 2013 to April 21, 2013. An OHCA case for the purpose of this study was defined as, “A patient who has an event of unresponsiveness and absence of breathing, outside the hospital setting” [7, 32, 33]. This was based on the EMS dispatch guidelines of American Heart Association (AHA) which advise that for all those patients who are unresponsive and not breathing normally, the dispatcher should recommend cardiopulmonary resuscitation (CPR) without pulse assessment. The omission of pulse check is because a witnessing bystander would take a longer time to do pulse checks and as such this assessment is often unreliable leading to type II error (false negative results). It also leads to a higher likelihood of not providing life-support interventions when patient actually needs it [34]. The same definition is also being used by emergency medical dispatchers worldwide to diagnose cardiac arrest on phone with a sensitivity of 70 % and specificity ranging from 95 to 99 % [7, 35]. The diagnosis was later confirmed by a physician; either in the ambulance or at an emergency department.

We excluded all patients who had a do-not-resuscitate status decided by the family at the time of arrest and arrests of non-cardiac etiology such as drug over dose, drowning, electrocution, asphyxia, respiratory disease or terminal illnesses. OHCA patients referred from other hospitals were also excluded to avoid duplication. Cause of arrest was assessed from the hospital records. In cases where this information was not available, each case was reviewed by a physician and a nurse and cause of arrest was assigned.

### Methods and measurements

Seventeen data collectors and a study coordinator were trained for data collection at all five hospitals. The data collectors provided coverage for twenty four hours at the selected hospital emergency departments. The study coordinator performed regular site visits and random quality checks to ensure completeness, accuracy and reliability of data collection. All data collectors received training on subject matter, data collection and therapeutic communication skills. The data was collected from three different sources; EMS personnel, hospital personnel and family members.

The questionnaire was developed using variables from standard data collection tools like the Cardiac Arrest Registry to Enhance Survival (CARES), Pan Asian Resuscitation Outcomes Study (PAROS) and American Heart Association (AHA) [36–38]. It was further modified according to the study objectives. (Please refer to Additional file 1 for questionnaire).

The questionnaire comprised of five different sections consisting of questions on: (1) General information (2) Arrest related information (3) Emergency medical services related factors (4) Hospital related factors and a separate section of questions to be asked from the family member if patient was brought by a personal/public transport. An important component in this study was accurate recording of time intervals. For this purpose, data collectors matched their watches with the ones being used at hospital and by EMS personnel to calculate the exact time intervals in minutes.

Face validation of the questionnaire was done by emergency medicine and public health experts. The questionnaire was originally developed in English and was translated into Urdu (local language). The translated version was back translated and no major discrepancies were found. The questionnaire was pretested in the emergency department of a major public sector hospital on 10 % of the calculated sample size.

In addition a small scale pilot of the project was also conducted at three of the study sites, which included one private and two public sector hospitals to test the design and overall feasibility of the project. The pilot project was conducted for three days in each hospital.

Questionnaire was further improved based on the results of questionnaire pretesting and pilot project. Major modifications to the questionnaire included; addition of questions about EMS clock synchronization with that of the dispatch center, questions about facilities present in the ambulance (CPR, defibrillator, life-saving medications and trained personnel) and minor changes in the language to bring clarity.

Double data entry was done to check for any discrepancies. 10 % of the entered data was further rechecked for accuracy. Epi Info version 2004 was used for data entry.

### Outcomes

OHCA survival at the end of emergency department stay was considered as the primary outcome and was defined as an OHCA patient being alive by the time he was shifted from the emergency department to an inpatient unit or any other hospital. Survival at the end of hospital stay was considered as a secondary outcome.

### Statistical analyses

The rate of survival at discharge from the emergency department was calculated from the number of patients surviving on discharge over the total number of OHCA patients. For the analysis and reporting of data points, we used Utstein style wherever possible [37, 39].

We examined characteristics between the groups of pre-hospital transportation using a chi-square test or a Fisher exact test. Continuous variables such as age, time to reach hospital and time to interventions were analyzed using one-way ANOVA [analysis of variance] or Kruskal-Wallis test. Results with a *p*-value of less than 0.05 were considered to be statistically significant. Furthermore, pair wise comparison was done using Tukey’s or Dunn’s test and a *p*-value of <0.017 between any pair was considered to be significant. Variables with missing information were merged to form a single variable (e.g. defibrillation, life-support medications and CPR were combined into the category of life-support interventions) to draw meaningful conclusions. Mean survival times between categories of transportation was compared using log-rank test with Kaplan Meier survival curves considering a *p*-value <0.05 to be significant. All analysis were carried out using SPSS (statistical package for social scientists version 19; IBM Corporation, NYC, US).

## Results

**Fig. 1:**
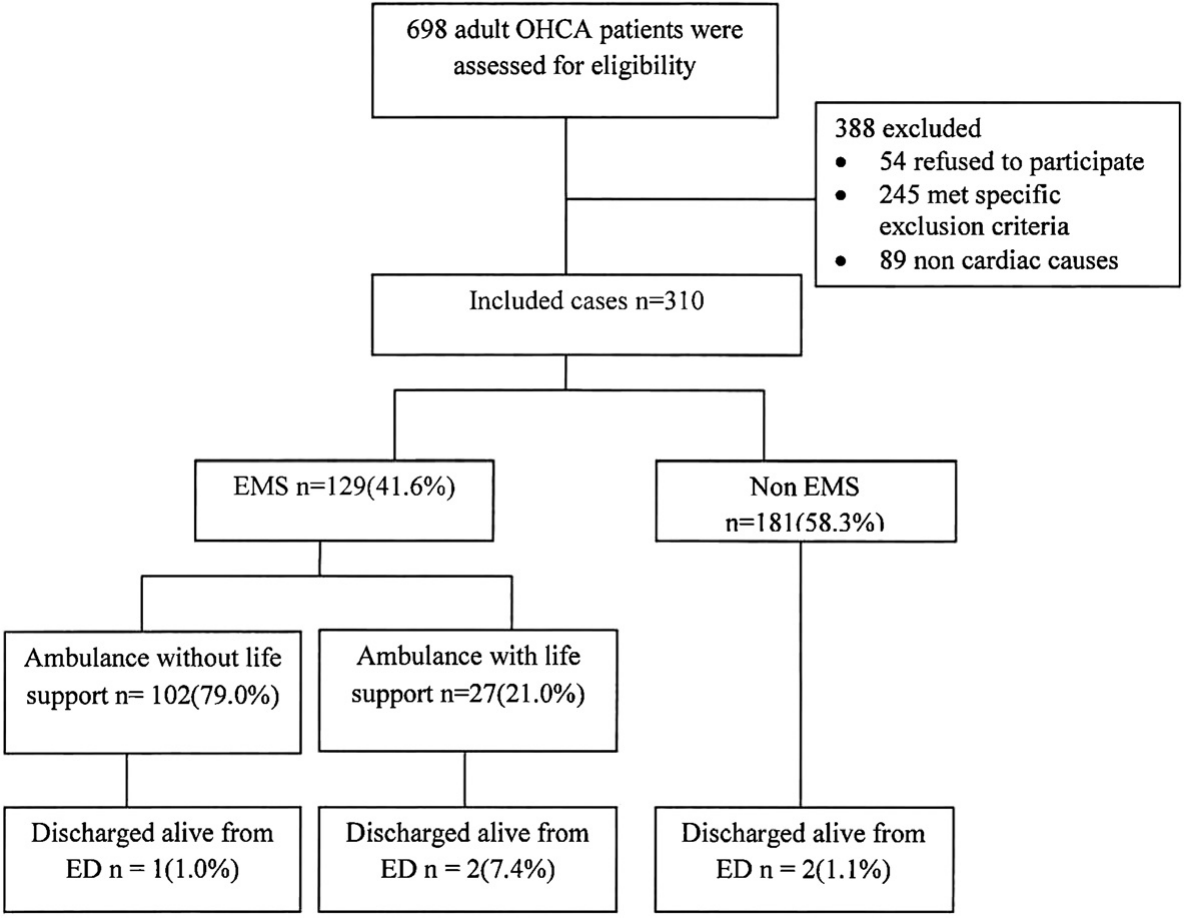
Flow diagram of a multicenter longitudinal cohort study. OHCA indicates out-of-hospital cardiac arrest, EMS indicates emergency medical services and ED indicates emergency department

**Fig. 2:**
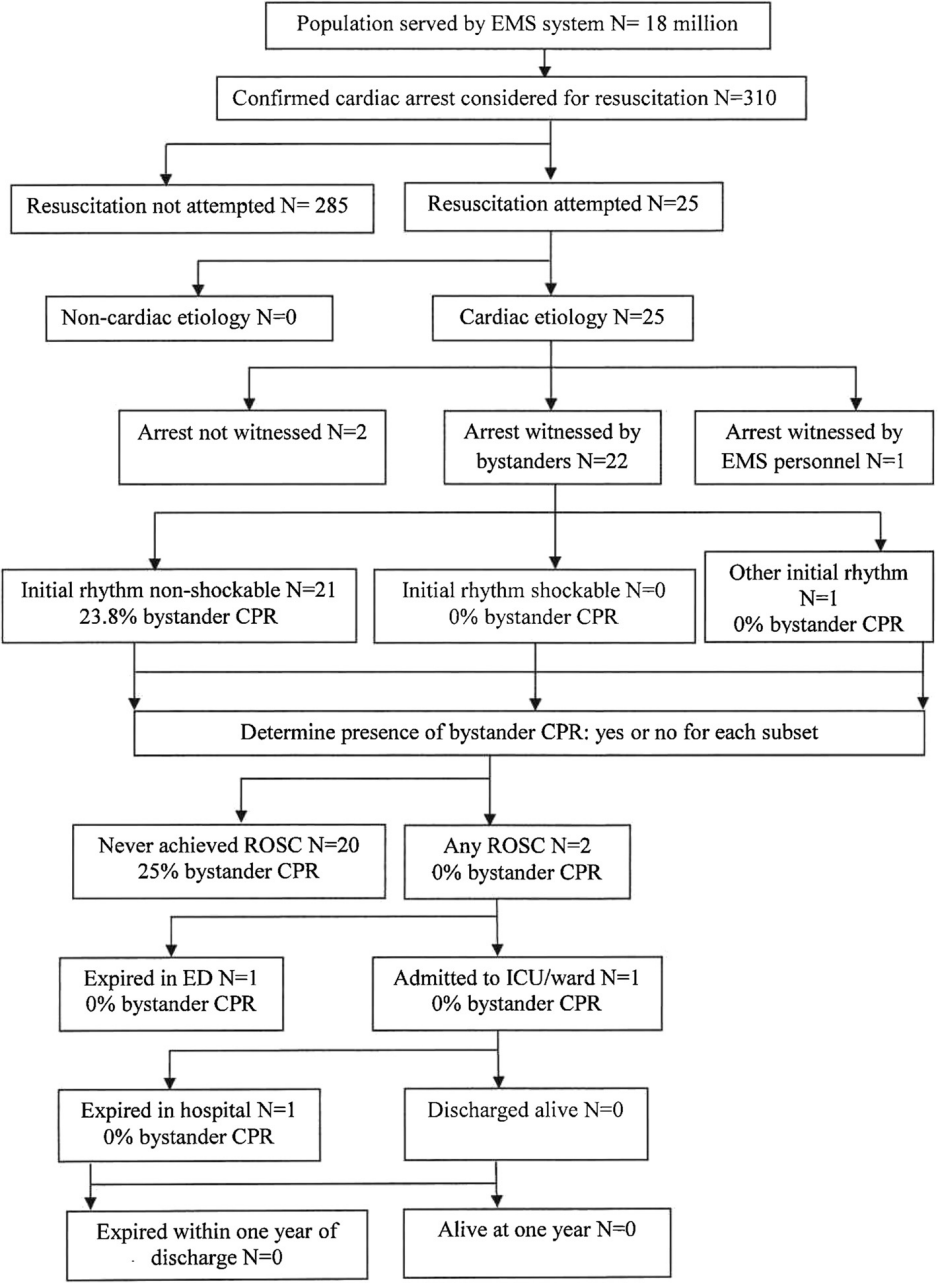
Utstein template for reporting data on out-of-hospital cardiac arrest

During the study period, a total of 698 patients presented to the study sites with out-of-hospital cardiac arrest, 54 family members refused to participate (12 %) (Fig. 1). Results are also summarized according to the Utstein style template (Fig. 2).

Our analysis includes 310 patients presenting with OHCA from the selected study sites. There were 105 women and 205 men with a mean age of 59.2 ± 15.1 years. We had a representative sample that included patients from all areas of Karachi. Majority of cases occurred in residences (77.7 %). 288 out of 310 (92.9 %) patients had a witnessed arrest, of which only 1.3 % of cases were witnessed by a health care worker. Patients utilizing ambulances with life-support interventions were older compared to patients presenting in other types of pre-hospital transportation. Gender, location of arrest and witness were not significantly different across different categories of pre-hospital transportation. However, a significantly higher percentage of patients utilizing ambulances with life-support facilities had ROSC (return of spontaneous circulation) during pre-hospital (3.7 %) and hospital settings (7.4 %). Overall, 8 patients had ROSC of which 5 (1.6 %) survived at the end of emergency department stay (Table 2). When these survivors were followed till the end of hospital stay, only two (0.6 %) patients were found to be alive and at two months follow-up after discharge, none of the patients were alive. Most of the patients having an ROSC died within a few hours of the event and the likely cause of death was hemodynamic instability rather than neurologic sequele. Only one of the patients had a documented evidence of neurological cause of death.

**Table 2:** Comparison of demographic and cardiac arrest related characteristics of 310 study participants

	No (%) of Patients				
Variables	All Patients	Non-EMS	EMS		*p*-value*
	(*n* = 310)	(*n* = 181)	Ambulance without any life-support (*n* = 102)	Ambulance with life-support (*n* = 27)	
Age (mean ± SE)	59.2 ± 15.1	58.2 ± 14.9	59.2 ± 15.5	65.3 ± 13.2	0.07
Gender					
Male	205 (66.1)	117 (64.6)	71 (69.6)	17 (63.0)	0.65
Female	105 (33.9)	64 (35.4)	31 (30.4)	10 (37.0)	
Comorbid conditions					0.09
Cardiac	179 (57.7)	106 (58.6)	58 (56.9)	15 (55.6)	
Non cardiac	11 (3.5)	4 (2.2)	7 (6.9)	0 (0)	
Both	23 (7.4)	13 (7.2)	5 (4.9)	5 (18.5)	
None	97 (31.3)	58 (32.0)	32 (31.4)	7 (25.9)	
Location of arrest					
Residence	241 (77.7)	146 (80.7)	76 (74.5)	19 (70.4)	0.30
Public area	69 (22.3)	35 (19.3)	26 (25.5)	8 (29.6)	
Witnessed arrest					
Yes	288 (92.9)	170 (93.9)	93 (91.2)	25 (92.6)	0.68
No	22 (7.1)	11 (6.1)	9 (8.8)	2 (7.4)	
Type of witness					
Layperson	284 (91.6)	170 (93.9)	90 (88.2)	24 (88.9)	0.15
Health care personnel	4 (1.3)	0 (0.0)	3 (2.9)	1 (3.7)	
None	22 (7.1)	11 (6.1)	9 (8.8)	2 (7.4)	
ROSC					
Pre-hospital	1 (0.3)	0 (0.0)	0 (0.0)	1 (3.7)	0.006
Hospital	7 (2.3)	4 (2.2)	1 (1.0)	2 (7.4)	
No ROSC	302 (97.4)	177 (97.8)	101 (99.0)	24 (88.9)	
Outcome^a^(end of ED stay)					
Alive	5 (1.6)	2 (1.1)	1 (1.0)	2 (7.4)	0.04
Dead	305 (98.4)	179 (98.9)	101 (99.0)	25 (92.6)	

*Abbreviations: EMS* emergency medical services, *SE* standard error, *ROSC* return of spontaneous circulation, *ED* emergency department

*Shows comparison between Non-EMS, EMS without any life-support interventions and EMS with life-support interventions

^a^Indicates outcomes on hospital admission

**Fig. 3:**
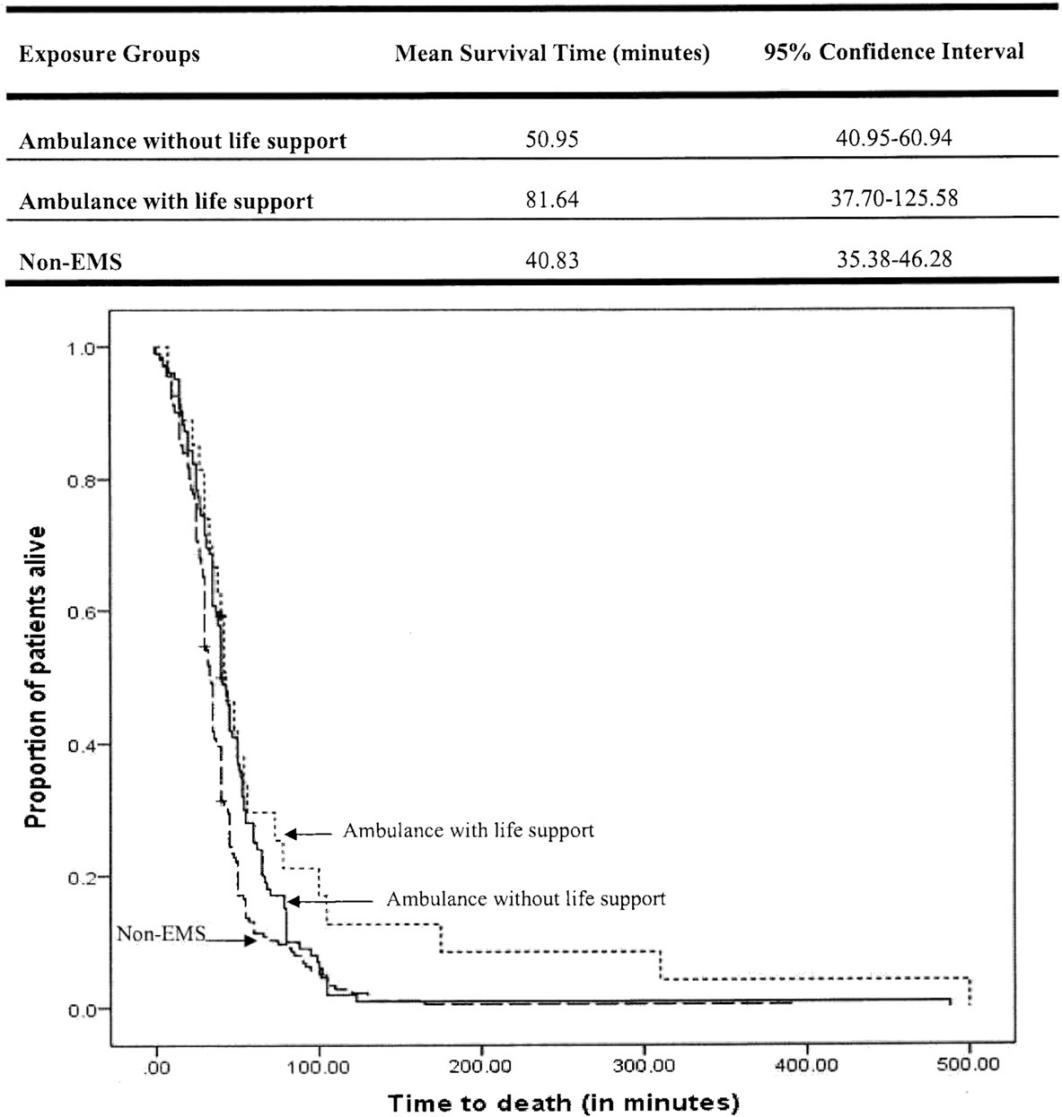
Kaplan Meier survival curves showing survival time by transportation status of cardiac arrest patients. Non-EMS indicates non-emergency medical services

Patients utilizing ambulances with life-support interventions survived for a significantly longer time compared to patients in the other two groups (log rank test, *p* = 0.002) (Fig. 3).

Time to first life-support intervention was calculated as the time from OHCA event to receiving CPR, shock or life-saving medication (whichever occurred first). The overall median time from arrest to receiving the first life-support intervention was 20.5 min (interquartile range [IQR] =12, 34.7), to first CPR was 20 min (IQR = 12.2, 37.7), to first shock was 23 min (IQR = 12, 35.2) and to first life-support medication was 25 min (IQR = 16, 42).

Although a higher percentage of the arrests were witnessed (92.9 %), only a very small percentage (2.3 %) of patients received bystander CPR. More than half of the patients were transported to the hospital via a non-EMS vehicle (private or public transport) (58.3 %) as compared to EMS (ambulances) transport and only a few patients received life-support interventions in pre-hospital settings (Table 3). Only 6 (1.9 %) patients, utilizing ambulances with life-support interventions, received epinephrine in the pre-hospital setting with a median (IQR) time of 15 min (10.5, 38). Artificial airway was used in 23 patients (7.4 %) in the emergency department. None of the patients received emergency angioplasty, coronary artery bypass graft or hypothermia.

**Table 3:** Comparison of life-support interventions received by cardiac arrest patients in Karachi, Pakistan (*N* = 310)

	No (%) of Patients				
Variables	All Patients	Non-EMS	EMS		*p*-value*
	(*n* = 310)	(*n* = 181)	Ambulance without life-support (*n* = 102)	Ambulance with life-support (*n* = 27)	
Time(min) to reach hospital					
Median (IQR)	25 (15, 35)	23 (15, 30)	30 (20, 40.2)	30 (18, 45)	<0.001
Life-support interventions					
Pre-hospital	25 (8.1)	2 (1.1)	2 (2.0)	21 (77.8)	
Hospital	48 (15.5)	34 (18.8)	12 (11.8)	2 (7.4)	<0.001
None	237 (76.5)	145 (80.1)	88 (86.3)	4 (14.8)	
Time to first intervention					
Median (IQR)	20.5 (12, 34.7)	18 (11, 30)	26 (17.2,51)	15 (9, 44)	0.01
Time to first CPR					
Median(IQR)	20 (12.2, 37.7)	19 (11.2, 28)	42 (22, 55)	16 (11, 44)	0.01
First rhythm					
Shockable	3 (1.0)	3 (1.7)	0 (0.0)	0 (0)	0.01
Non shockable	277 (89.4)	168 (92.8)	84 (82.4)	25 (92.6)	
Not recorded	30 (9.7)	10 (5.5)	18 (17.6)	2 (7.4)	
CPR					
Pre-hospital	23 (7.4)	2 (1.1)	2 (2.0)	19 (70.4)	<0.001
Hospital	48 (15.5)	33 (18.2)	11 (10.8)	4 (14.8)	
None	239 (77.1)	146 (80.7)	89 (87.3)	4 (14.8)	
Defibrillation					
Pre-hospital	1 (0.3)	0 (0.0)	0 (0.0)	1 (3.7)	0.02
Hospital	95 (30.6)	56 (30.9)	33 (32.4)	6 (22.2)	
None	214 (69.0)	125 (69.1)	69 (67.6)	20 (74.1)	

*Abbreviations: EMS* emergency medical services, *IQR* interquartile range, *CPR* cardiopulmonary resuscitation

*Shows comparison between Non-EMS, EMS without any life-support interventions and EMS with life-support interventions

Some important differences were observed while analyzing the same dataset subdivided across categories of transportation. Patients utilizing non-EMS transport reached the hospital earlier as compared to patients utilizing EMS with or without life-support interventions (23 min vs. 30 min). These patients also had a shorter time to receive first life-support intervention (non-EMS:18 min vs. EMS: 23 min). However, if the same variable is compared over the three transportation groups, ambulances with life-support interventions had the shortest median time to life-support interventions of 15 min (IQR = 9, 44) compared to 26 min for an ambulance without life-support interventions (IQR = 17.2, 51) and 18 min for a private transportation (IQR = 11, 30) (*p* = 0.01). Dunn’s pairwise comparison for the time to reach hospital showed that the time was significantly shorter for non-EMS as compared to ambulances with life-support interventions (*p* = 0.011) as well as those without life-support interventions (*p* < 0.001). Time to first intervention was significantly different between ambulances without life-support interventions and non-EMS (*p* = 0.004).

A small proportion of the patients had a shockable rhythm on first assessment; 0 % in EMS whereas 1.7 % in non-EMS (*p* = 0.005). In contrast, a higher percentage of patients received defibrillation, mainly in hospital settings (30.2 % in EMS vs. 30.9 % in non-EMS) (*p* = 0.49). The majority of OHCA patients did not receive CPR at all with the highest percentage of CPR and shortest time to receive CPR being observed in the category of patients utilizing ambulances with life-support interventions (Table 3). Dunn’s pairwise comparison showed significant differences in time to CPR between ambulances with life-support interventions versus those without life- support interventions (*p* = 0.015). It was also significantly different between non-EMS and ambulances without life-support interventions (*p* = 0.006).

## Discussion

This is the first city wide study for OHCA, assessing its outcomes in Karachi, Pakistan. We found zero percent survival rate for patients with cardiac arrest two months after the event, <2 % survival rate at the end of emergency department stay and less than 1 % at hospital discharge. We also found that bystander CPR was rarely done, resulting in large delays in the first attempted CPR. Ambulances with life-support interventions provided more pre-hospital CPR (70.4 %) than any other groups (non-EMS = 1.1 % and ambulance without life-support interventions = 2.0 %) with significantly shorter median time to first CPR (16 min for ambulance with life-support, 19 min for non-EMS and 42 min for ambulance without life-support interventions, *p* = 0.01). Patients transported through life-support ambulances had a higher likelihood of surviving to hospital admission, but there was no difference in the eventual outcome irrespective of the type and timing of pre-hospital response. Nevertheless, an important observation is the difference in survival times. Being transported by ambulance with life-support interventions provides additional minutes for life-saving interventions as compared to those utilizing non-EMS transportation (81.6 min vs. 40.8 min, *p* = 0.002).

Survival rates reported in this study are lower than any international or national study, including earlier studies from Pakistan. A systematic review of over 60 studies from high income countries of North America, Europe, Australia and Asia found an average survival to hospital discharge of about 7 % (range 0.6 to 25 %) [6]. Studies from developing countries have shown varying survival from as low as 0 % in Mexico to 2 % in Islamabad, Pakistan and 11 % in Karachi, Pakistan. The study from Mexico was based on EMS data while the study from Karachi observed 56 OHCA cases and the one from Islamabad reviewed 50 OHCA cases presenting to a single private sector hospital. Both of the studies from Pakistan reported comparably higher survival rates. These studies have limited generalizability due to smaller sample sizes and using data from a single hospital which is not representative of the diverse general population of the country [16–18]. In our study, the private hospital from the study conducted in Karachi did not show any survivors either.

In our study, despite the majority of arrests being witnessed (92.9 %), the percentage of bystander CPR was very small (2.3 %, *n* = 7). Of these, only 3 were dispatch assisted CPR. Literature reports beneficial effects of bystander CPR [40]. Lack of bystander CPR in our study resulted in a delay of 20 min before CPR was initiated; a time span much longer than the recommended 3-4 min [16, 41]. In our cohort, although the survivors had a shorter time to first life-support intervention as compared to non-survivors (13.7 min vs. 30.5 min), this time was still long enough to unleash irreversible metabolic processes post cardiac arrest, which explains why patients who were brought alive to the ED did not survive on follow-up. Also a small number of patients had shockable rhythm on first assessment (1.0 %) but were not defibrillated in due time.

The availability of pre-hospital emergency medical care is a norm in most developed countries. Considered as an important part of public health system, modern EMS has well defined training and performance standards [42]. Although, the literature comparing survival benefits of EMS vs. non-EMS has shown varying results; cardiac arrest is one area where EMS makes the largest difference in patient outcomes [17, 18, 41, 43, 44].

In developing countries like Pakistan, EMS is still not considered a medical intervention, rather a quick way of transportation to and from hospital [24, 31]. This study highlights that a formal EMS may have some benefits for short term survival in Karachi. Alternatively, an ambulance without any pre-hospital care may be worse than regular private transportation.

The results from our data suggest that strengthening the health system responsible for emergency care and transportation has a potential to save a large number of lives. Beginning with the public training programs to train bystanders, the first part of the chain of survival needs to be strengthened in Karachi. This could be achieved through programs embedded in the schools curricula and workplace settings. Dispatch instructions can also be helpful in increasing the percentage of bystander CPR. In addition, the quality of care in EMS system also needs considerable improvement, with every EMS vehicle having trained personnel and equipment to provide basic and advanced life-support interventions. In the current scenario most of the ambulance services in Karachi are private transport vehicles with no life-saving drugs or equipments on board except oxygen cylinders. In the absence of government funding, private organizations should pool resources for improving the quality of pre-hospital care and expanding EMS services. Instead of several different organizations working in isolation and providing varying quality of care, a single network of ambulances should be developed to provide pre-hospital care according to international standards. Establishment of a universal access number, a central dispatch system and improving post cardiac arrest care in the hospitals will also improve the overall chain of survival in the city. In addition, public locations with a higher incidence of OHCA should be identified and facilities for public access defibrillator must be provided to reduce time intervals in the chain of survival. A state-wide OHCA registry also needs to be developed to monitor the outcomes of OHCA in general and in response to interventions.

Our study had certain limitations. First, the study was conducted in 5 major teaching hospitals in Karachi which represent a substantial proportion of, but not all patients presenting to hospitals with cardiac arrest. Secondly, due to lack of survivors and lack of pre-hospital interventions we could not ascertain what factors are more important for survival than others. Third, being an observational study though prospective, it could have been biased by the number of patient families who refused to participate. Fourth, we did not intend to measure hospital care through this study, where significant findings were; completely incorrect practice of delivering shocks in patients with non-shockable rhythms and a small frequency of CPR in hospital emergency departments. Last but not least, due to lack of trained EMS personnel and most arrests being assessed by lay persons, we developed the operational definition of diagnosing cardiac arrest based on recommendations by AHA for Lay responders. According to this definition, absence of response and breathing is diagnosed as cardiac arrest without any pulse assessment. This, we believe might have misdiagnosed cardiac arrests in some cases and included respiratory arrests as well.

## Conclusion

In summary, we found a 0 % survival rate for OHCA in Karachi. Delay in CPR and other life-saving interventions were some of the important observations. This was mainly due to lack of bystander and dispatch assisted CPR, use of public/private vehicles for pre-hospital transportation of OHCA patients instead of ambulances and weaker pre-hospital systems. Additional studies are required to identify factors associated with the survival of OHCA patients. Poor survival in this setting emphasizes the need to standardize EMS systems, initiate public awareness and training programs, and improve post-arrest care to strengthen the links in the chain of survival.

### Abbreviations

AHA, American Heart Association; ANOVA, analysis of variance; CARES, Cardiac Arrest Registry to Enhance Survival; CPR, cardiopulmonary resuscitation; ED, emergency department; EMS, emergency medical services; IQR, interquartile range; OHCA, out of hospital cardiac arrest; PAROS, Pan Asian Resuscitation Outcomes Study; ROSC, return of spontaneous circulation; SPSS, statistical package for social scientists

## Additional file

Additional file 1: Study Questionnaire. (PDF 287 kb)
