# Supplementary material for: Epidemiology and outcomes of out-of-hospital cardiac arrest in a developing country-a multicenter cohort study
Source: BMC Emerg Med. 2016 Jul 28;16:28. doi: 10.1186/s12873-016-0093-2 (PMC4963996; doi:10.1186/s12873-016-0093-2)
Supplement: Additional file 1: — Study Questionnaire. (PDF 287 kb) [file 12873_2016_93_MOESM1_ESM.pdf]

Additional file I- Questionnaire

Hospital name (code): ________

Form Number: _____________

Burden of Out of Hospital Cardiac arrest and the association of type of transportation with survival

Questionnaire

Section A: General Information

1) Form Number: _ _ _ _

2) Hospital Name: _____________

☐ASH =1 ☐ CHK =2

☐ JPMC =3 ☐ AKUH =4

☐KIHD =5

3) Name of the Relative (responder): _______________

4) Relative's Contact Number: __________________

5) Patient's hospital Identification number: _ _ -_ _ -_ _

6) Patient's full name: _________________

7) Time of calling the ambulance :__________(HH:MM) (to be asked from person accompanying the patient)

Hospital name (code):________

Form Number: _____________

8) Patient's age (in years):_________________

9) Patient's Date of birth: ☐☐/☐☐/☐☐ (DD/MM/YY)

10) Gender:

☐ Male =1 ☐ Female =2

11) Name of the data collector: _______________

12) Date of data collection☐ ☐/☐ ☐ / ☐ ☐ (DD/MM/YY)

Section B: Cardiac Arrest Related Information (to be extracted from hospital records)

13) Date of arrest: ☐☐ /☐ ☐ /☐ ☐ (DD/MM/YY)

14) Time of arrest :_____________(to be recorded in 24 hour format: HH:MM)

15) Location of Arrest (please tick one)

☐ Residence/home =1

☐ Physician's clinic=2

☐ Workplace =3

☐ On Street =4

☐ In ambulance=5

☐ Other :_____________(*please specify*)

Hospital name (code): ________

Form Number: ____________

16) Area of event: ______________ (where patient was found)

17) Cause of Arrest:

☐Cardiac cause =1 ☐Trauma/injury=2 ☐ Respiratory disease=3

☐Electric shock=4 ☐Drowning=5 ☐Drug overdose=6

☐Asphyxia=7 ☐Any other=8_________ (*please specify*)

18) Was anyone present with the patient when the event of cardiac arrest occurred?

☐Yes =1 (go to question 19) ☐No =2 (go to question number 20)

19) Who was present with patient at the time of event?

☐Bystander layperson=1

☐Health care professional (not part of EMS team) =2

☐Relative=3

☐Ambulance staff=4

20) Comorbids (Mark all that is applicable)

☐Hypertension =1 ☐Diabetes =2 ☐Heart disease=3

☐Hypercholesterolemia =4 ☐ Cancer=5 ☐Paralysis=6 ☐Renal disease=7

☐ Others=8 :____________(*please specify*) ☐ Not known=9

21) Patient's mode of transportation to hospital

☐ Edhi ambulance = 1 (please fill section C) ☐ Aman Ambulance = 2 (please fill Section C)

☐ Any other ambulance=3_______________ (*please specify*) (please fill Section C)

☐ Personal transport =4(go to Section D) ☐ Public transport (e.g. rickshaw, taxi etc.) =5 (go to section D)

Hospital name (code): ________

Form Number: ______________

Section C: Emergency Medical Services (EMS)/Ambulance related factors (to be extracted from EMS records/inquiring EMS personnel)

22) Type of ambulance

☐ Ambulance with only driver=1

☐ Ambulance with CPR (cardio pulmonary resuscitation) facility=2

☐ Ambulance with CPR and AED (Automated External Defibrillator) facility=3

☐ Ambulance with facilities of CPR, AED and Lifesaving drugs =4

23) How does your ambulance service keep record of time?

☐Exact time (minutes and seconds)=1 ☐ Approximate time =2

24) Current time (according to hospital clock) :______________(to be recorded in 24 hour format: HH:MM)

25) Current time (according to ambulance personnel clock) :__________(to be recorded in 24 hour format: HH:MM)

(Question numbers 24 and 25 are for the purpose of calculating time difference so that accurate time differences can be calculated)

26) Did ambulance personnel match time according to his watch with the clock at the dispatch center?

☐Yes =1 ☐No =2

27) Time of call to ambulance dispatch Centre: __________(HH:MM) (will be inquired from their office)

28) Time of call to ambulance :__________(HH:MM) (to be inquired from ambulance personnel)

Hospital name (code):________

Form Number: ____________

29) Time of ambulance arrival at the scene :__________(HH:MM)

30) Time of ambulance departure from the scene :__________(HH:MM)

31) Was anyone advised, on phone by the Ambulance staff, to do patient's CPR before ambulance arrival?

☐Yes =1 ☐No =2

32) Was CPR already being provided when Ambulance reached at the location?

☐Yes =1 (go to question 33) ☐ No =2(go to question 34)

33) Who provided CPR?

☐Bystander layperson=1

☐Health care professional (not part of EMS team) =2

☐Relative=3

☐Ambulance staff=4

34) First Rhythm

☐ Shockable (ventricular tachycardia, ventricular fibrillation) =1

☐ Non-Shockable (pulseless electrical activity, asystole) =2

☐ Could not be recorded =3

35) Did Ambulance services personnel provide any life-saving interventions? (E.g. CPR, Shock, lifesaving medications)?

☐Yes (complete questions 36-43) =1 ☐No (go to Q# 44)=2

Hospital name (code):________

Form Number: _____________

36) Was CPR provided by the Ambulance team when they reached the location of arrest?

☐Yes (go to Q# 37) =1 ☐No (go to Q# 38) =2

37) Time when CPR was initiated___________ (HH:MM)

38) Was shock (defibrillation) administered to patient?

☐Yes =1 (go to Q# 39) ☐ No (go to Q# 40)=2

39) Time of shock_____________ (HH:MM)

40) Was Life support Medication Administered?

☐Yes =1(go to Q# 41) ☐No (go to Q# 43) =2

41) What life saving drugs were given to patient

☐Epinephrine=1 ☐Atropine=2 ☐ Amiodarone=3 ☐Bicarbonate=4

☐Lidocaine =5 ☐ Dextrose=6 ☐ Any other____________ *(please specify)*

42) Time when first Life support Medication was given__________ (HH:MM)

43) What time was CPR stopped? ___________ (HH:MM)

44) What was the reason to stop/ not initiate CPR?

☐Family decided code as DNR=1 ☐ Return of spontaneous circulation=2

☐Death declared by physician=3 ☐ Any other reason_________ *(please specify)*

Hospital name (code):________

Form Number: _____________

45) Was there a return of pulse? (Spontaneous circulation)

☐ Yes (go to Q# 46) =1 ☐ No (go to Q# 47)=2

46) Time of return of Pulse_____________(HH:MM)

47) Time when ambulance reached hospital_________ (HH:MM)

<u>Section D: Hospital Related Factors (to be extracted from hospital records)</u>

48) Date when patient reached the hospital ☐☐ /☐☐ /☐☐ (DD/MM/YY)

49) Time when the patient reached Hospital_______________ (HH:MM) (*observed by data collector*)

50) Time when hospital staff assessed the patient __________ (HH:MM) (*observed by data collector*)

51) Was patient breathing on arrival at hospital?

☐ Yes =1 ☐ No =2

52) Did patient have pulse on arrival at hospital?

☐ Yes =1 ☐ No =2

53) First recorded cardiac rhythm in ED

☐ Shockable (ventricular tachycardia, ventricular fibrillation) =1

☐ Non shockable (asystole, pulseless electrical activity) =2

☐ Could not be recorded =3

Hospital name (code): ________

Form Number: ______________

54) Patient's outcome on arrival at ED?

☐ Alive =1

☐ Declared dead (DOA) =2

55) Was CPR provided to the patient in ED?

☐Yes (go to Q#56)=1 ☐No (go to Q# 57) =2

56) Time when CPR was initiated___________ (HH:MM)

57) Was shock (defibrillation) administered to patient?

☐ Yes =1 (go to Q# 58) ☐No (go to Q# 59) =2

58) Time of shock_____________ (HH:MM)

59) Was Life support Medication Administered?

☐Yes =1 (go to Q#60) ☐No (go to Q# 62)=2

60) What life saving drugs were given to patient

☐Epinephrine=1 ☐ Atropine=2 ☐ Amiodarone=3 ☐ Bicarbonate=4

☐Lidocaine =5 ☐ Dextrose=6 ☐ Any other____________ (*please specify*)

61) Time when first Life support Medication was administered__________ (HH:MM)

62) What time was CPR stopped? ___________ (HH:MM)

63) What was the reason to stop/ not initiate CPR?

☐Family decided code as DNR=1 ☐ Return of spontaneous circulation=2

☐Death declared by physician=3 ☐ Any other reason_________ (*please specify*)

64) Was there a return of pulse (spontaneous circulation)?

☐Yes (go to Q# 65) =1 ☐ No (go to Q#66)=2

65) Time of return of spontaneous circulation____________ (HH:MM)

66) To maintain patient's airway, was any artificial airway used?

☐ Yes (go to Q# 67) =1 ☐ No (go to Q#68) =2

67) What type of artificial airway was used?

- ☐ Oral Endotracheal tube=1
- ☐ Nasal Endotracheal tube=2
- ☐ Laryngeal Mask Airway=3
- ☐ Any other___________(*please specify*)

68) Was any of the following treatment given to the patient?

- ☐ Emergency Angioplasty=1
- ☐ Emergency coronary Artery Bypass Graft=2
- ☐ Hypothermia=3
- ☐ Any other___________(*please Specify*)

69) Patient's Condition at the end of ED stay

☐ Alive (Shifted from ED) =1

☐ Alive but left before recovery (Leave against medical advice) =2

☐ Declared dead=3

Hospital name (code):________

Form Number: _____________

70) Date and time of patient's end of ED stay

Date: ☐☐/☐☐ /☐☐ (DD/MM/YY) (hospital records)

Time :____________(HH:MM)

71) Patient's condition at the end of hospital stay

☐Alive on discharge =1

☐Alive but left before recovery (Leave against medical advice/shifted to other hospital) =2

☐Declared dead =3

72) Date and time of patient's end of hospital stay

Date: ☐☐/☐☐ /☐☐ (DD/MM/YY) (hospital records)

Time :____________(HH:MM)

73) Was patient's Cerebral Performance Category Score checked at the end of hospital stay?

☐Yes (go to Q# 74) =1 ☐No (go to next section) =2

74) What was patient's Cerebral Performance category score (CPC) at the end of hospital stay?

____

Section E: To be filled only if patient arrived via personal/public transport (Non-EMS)

To be asked from patient's relative/accompanying person

75) Was CPR provided to patient before reaching to the hospital?

☐Yes (complete Q# 76 to Q#79) =1

☐No (go to Q# 80) =2

Hospital name (code): ________

Form Number: ____________

76) Who provided CPR?

☐ Bystander layperson=1

☐ Health care professional (not part of EMS team) =2

☐ Relative=3

☐ Ambulance staff=4

77) Time when CPR was initiated : ________ (HH:MM)

78) Time when CPR was stopped: ________ (HH:MM)

79) What was the reason to stop CPR?

☐ Family decided code as DNR=1 ☐ Return of spontaneous circulation=2

☐ Death declared by physician=3 ☐ Any other reason ________ (*please specify*)

80) At what time did you get transport to bring patient to the hospital? __________ (HH:MM)

Details of the Incident

____
____
____
____

Signature of the interviewer: ____________________
